# Health economics-based verification of functional myocardial ischemia evaluation of stable coronary artery disease in Japan: A long-term longitudinal study using propensity score matching

**DOI:** 10.1007/s12350-020-02502-9

**Published:** 2021-01-18

**Authors:** Tomoyuki Takura, Hiroyoshi Yokoi, Nobuhiro Tanaka, Naoya Matsumoto, Eri Yoshida, Tomoaki Nakata, J-CONCIOUS Investigators

**Affiliations:** 1 Department of Healthcare Economics and Health Policy, Graduate School of Medicine, The University of Tokyo, 7-3-1, Hongo, Bunkyo-ku, Tokyo, 113-8655 Japan; 2 Cardiovascular Center, Fukuoka Sanno Hospital, International University of Health and Welfare, Fukuoka, Japan; 3 Department of Cardiology, Tokyo Medical University Hachioji Medical Center, Tokyo, Japan; 4 Department of Cardiology, Nihon University Hospital, Tokyo, Japan; 5 Nihon Medi-Physics Co., Ltd., Tokyo, Japan; 6 Hakodate Goryoukaku Hospital, Hakodate, Japan

**Keywords:** Stable coronary artery disease, Cost-effectiveness analysis, Functional myocardial ischemic evaluation, Elective percutaneous coronary intervention, Propensity score, Major adverse cardiovascular event, Medical cost

## Abstract

**Background:**

The procedural numbers and medical costs of percutaneous coronary intervention (PCI), mainly elective PCI, have been increasing in Japan. Owing to increased interest in the appropriateness of coronary revascularization, we conducted this medical economics-based evaluation of testing and diagnosis of stable coronary artery disease (CAD).

**Methods and Results:**

We reviewed patients’ medical insurance data to identify stable CAD patients who underwent coronary computed tomography angiography, cardiac single-photon emission computed tomography, coronary angiography, or fractional flow reserve. Subjects were divided into anatomical and functional evaluation groups according to the modality of testing, and background factors were matched by propensity score. The endpoints were major adverse cardiovascular events (MACE), life years (LYs), medical costs, and cost-effectiveness analysis (CEA). The observations were performed for 36 months. MACE, medical costs, and CEA of the functional group in the overall category were trending to be better than the anatomical group (MACE, *P* = .051; medical costs: 3,105 US$ vs 4,430 US$, *P* = .007; CEA: 2,431 US$/LY vs 2,902 US$/LY, *P* = .043).

**Conclusions:**

The functional evaluation approach improved long-term clinical outcomes and reduced cumulative medical costs. As a result, the modality composition of functional myocardial ischemia evaluation was demonstrated to offer superior cost-effectiveness in stable CAD.

**Supplementary Information:**

The online version of this article (10.1007/s12350-020-02502-9) contains supplementary material, which is available to authorized users.

## Introduction

As a result of the remarkable increase in the aging population and lifestyle habit changes in Japan, coronary revascularization was performed on 296,743 patients in 2018, or 234.7 cases/year per 100,000 population (as of October 2018).^1^ Of the 278,285 percutaneous coronary intervention (PCI) procedures performed in 2018, elective PCI indicated for patients with stable coronary artery disease (CAD) accounted for a large proportion (72.4%) of these procedures. Over a single 5-year period (2012–2017), the number of PCI procedures increased by 6.9% annually, with medical costs for cardiovascular procedures, including coronary revascularization, accounting for 19.7% (2016) of the national medical costs in Japan.^2^,^3^ Appropriate use of PCI, therefore, has been noted as one of the urgent issues to be resolved, not only from the viewpoint of reducing the disease burden, but also from a socioeconomic standpoint. Clinical indications and therapeutic outcomes, as well as cost-effectiveness, are garnering attention, with elective PCI as a topic of particular interest.

Investigations of CAD treatment strategies showed that the degree of anatomical coronary stenosis and level of severity of functional ischemia do not always coincide.^4^–^7^ In particular, an appropriate therapeutic strategy for patients with moderate stenosis cannot necessarily be selected on the basis of the anatomical degree of coronary stenosis alone.^5^,^6^,^8^,^9^ Revascularization has been shown to improve prognosis in patients with progressive or high-risk myocardial ischemia at or above a set level in cases affected by acute coronary syndrome (ACS) or stable CAD.^6^,^7^,^9^,^10^ On the other hand, favorable clinical outcomes can be maintained for a certain number of stable CAD cases by appropriate outpatient management with risk-based optimal medical therapy (OMT) alone.^5^,^7^,^9^,^11^ The anatomical evaluation of coronary narrowing with conventional coronary angiography (CAG) is insufficient for selecting appropriate treatment for stable angina pectoris patients. In addition, a comprehensive evaluation based on plaque lesion morphology and fractional flow reserve (FFR) testing is closely associated with clinical risks and outcomes.^12^ ACS risk and related cardiac mortality tend to increase significantly in relation to the level of residual myocardial ischemia manifested during follow-up.^13^–^15^ Thus, the evaluation of the severity of functional ischemia is critical for the diagnosis and risk assessment of stable CAD; for the selection of invasive diagnostic and therapeutic approaches including PCI; and for the prediction of clinical outcomes.

There are a small number of medical economics reports related to testing and PCI for patients with stable CAD. Coronary FFR-guided PCI is shown, not only to improve prognosis, but also to shorten hospital stays together with reducing the amount of contrast medium and number of placed stents.^9^ The FAME study, which included ACS patients, showed that the FFR-guided strategy can reduce the major cardiac event rates after 2 years and the number of patients when compared to CAG-guided strategy.^5^,^16^ Performing PCI on stenotic lesions with no functional ischemia does not result in a favorable prognosis.^17^ In particular, performing PCI on lesions that are negative for functional myocardial ischemia based on FFR is considered inappropriate because it does not improve the prognosis.^6^,^18^ Furthermore, the socioeconomic costs of such advanced testing are high. For these reasons, a cost-effectiveness analysis (CEA) is important to reveal the clinical benefits gained and medical resources expended for establishing the appropriate selection of a diagnostic procedure and treatment strategy in patients with stable CAD.

From 2019, CEA of pharmaceuticals and medical devices has been fully implemented in the Japanese medical insurance system. Despite previous reports of CEA for elective PCI in Japan,^19^,^20^ there are very few studies which describe CEA in association with diagnostic modalities and actual diagnostic grounds for coronary revascularization. Real-world longitudinal studies that compare several modalities for testing and diagnosis are particularly rare worldwide. In addition, assessment of the functional severity of myocardial ischemia became a billing requirement for elective PCI under the 2018 revision of medical fees by the Japanese Government’s National Health Insurance. Therefore, we designed this study on the medical economics-based evaluation using CEA in relation to advanced diagnostic modalities, treatment options, and outcomes in patients with stable CAD, particularly focusing on the difference between functional and anatomical evaluations.

## Methods

### Study Design

This retrospective cohort study used a large database. The data source was the medical service data examined by a public specialized organization (Social Insurance Medical Fee Payment Fund) in accordance with the format stipulated by the Japanese government’s Ministry of Health, Labor and Welfare (MHLW Notification: Vol. 0831 No. 1). From this data source, we selected medical economic big data (TheBD: The Tokyo University Health Economy Big Data), which included medical service bills gathered from public insurers (including health insurance societies of companies) throughout Japan in this study. These data have only been recently available for research in 2019. Data from 7 million insured patients were gathered. This database is updated every 6 months and is linked in chronological order by management ID. During each biannual update, transfer of insured persons will be managed, and adjustments will be made according to medical facilities relocations. As for the sample composition by year, 2016 was the largest with 22.1% of the total (reference table: Table S1). In addition, medical information was 6.18 million, and dispensing information was 6.20 million (including duplication). The patient-based hospitalization rate was 13.5% (including duplications) and the average male ratio for all years was 46.8%. We examined the medical service received at hospitals, clinics, and pharmacies between April 2012 and March 2019 based on the therapy performed (including testing/diagnosis, pharmacotherapy, treatment/surgery, hospitalized recuperation, rehabilitation, outpatient treatment); outcomes (including death and hospital transfer); and medical costs and chronologically linked subjects with unified IDs. This medical economics study, which applied big data, was given comprehensive approval on March 2019 by the institutional review board of the University of Tokyo Hospital (screening no.: 2018167NI). This study was carried out in accordance with the RECORD statement,^21^ and the study plan was preregistered (registration no.: UMIN000040282). As we used database records for analysis, the need for informed consent was waived (opt-out format). Owing to the sensitive nature of the data collected for this study, data which support the findings of this study are available from the corresponding author upon reasonable request.

### Study Subjects

**Figure 1:**
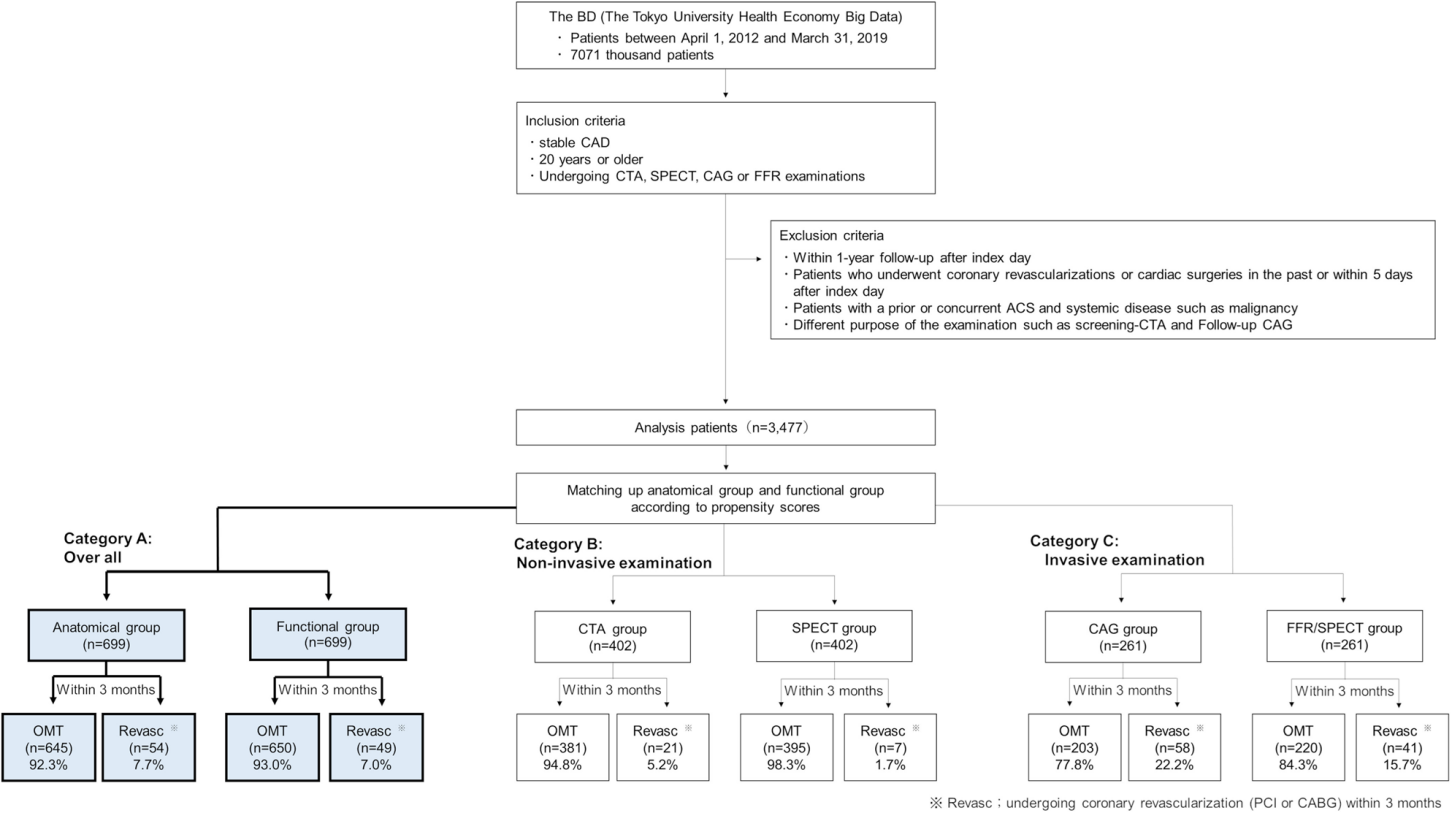
Case data setting procedure and analysis group classification results (sample size composition). Subjects were allocated into the anatomical and functional groups based on a propensity score

We identified stable CAD in the subjects for analysis from the database using information, such as age when treatment was received, main disease (International Classification of Diseases 10^th^ Revision: ICD-10), and treatment history (Figure 1). In this study, only patients with a disease code indicating stable CAD was extracted from the range of ICD-10 code I11.0 through I50.9 as the eligibility criteria. In addition, the subjects were adults aged 20 years or older who were undergoing testing or diagnosis related to the degree of anatomical coronary stenosis or functional ischemia severity for the first time. In consideration to the study purpose and analysis conditions, the tests that were relatively expensive and widely used as clinical practice were evaluated as medical technology. Based on the above, we selected coronary computed tomography angiography (CTA), cardiac single-photon emission computed tomography (SPECT), CAG, and FFR as evaluation tests. The index day was the first day on which analyzed CTA, SPECT, CAG, or FFR testing were performed. Subjects were followed up for at least 1 year (excluding cases of mortality).

The exclusion criteria were as follows: prior coronary revascularization, history of ACS within 1 year before the index day, and any latent risk factors that could affect the evaluation of cardiac disease diagnosis and treatment, including cardiac-related surgery, assisted circulation, or arrhythmia device implantation. We also excluded cases thought to have a low risk of cardiac disease. These included patients undergoing CTA alone for whom stress electrocardiogram (ECG) or stress echocardiography had not been performed within 1 year before the index day, and patients not treated with antiplatelet agents within 6 months after CAG (excluding cases considered to have no coronary stenosis). Patients undergoing CAG alone who had undergone the same testing two or more times were excluded for the purpose of excluding follow-up CAG. To rule out cases of urgency, we excluded cases in which coronary revascularization was performed within 5 days of the index day, which corresponds to emergency coronary revascularization. Patients with concurrent systemic diseases, including malignant tumors or Kawasaki disease (sequela), as well as general injuries, were excluded because these factors could affect prognosis and medical costs. After excluding cases from the subjects according to the aforementioned criteria, we included 3,477 cases in our comparison.

### Study Evaluation Methods

We compared the effects of the selection (composition) of various modalities affecting treatment prognosis and medical costs for 3,477 cases. We broadly divided subjects into anatomical and functional evaluation groups and also categorized subjects according to whether they entered the catheterization laboratory.

Subjects were divided into the following groups according to the modality. Anatomical group: subjects who only underwent CTA (CTA group) and those who underwent only CAG or, in some cases, CAG and CTA (CAG group); Functional group: those who underwent only SPECT or, in some cases, SPECT and CTA (SPECT group), and those who underwent either CAG and FFR or SPECT and CAG (FFR/SPECT group). Category A included the entire cohort, Category B consisted of groups excluding cardiac catheter testing (CTA and SPECT groups), and Category C consisted of groups including cardiac catheter testing (CAG and FFR/SPECT groups). Then each category of A, B, C was divided into anatomical and functional groups and compared (Figure 1).

**Figure 2:**
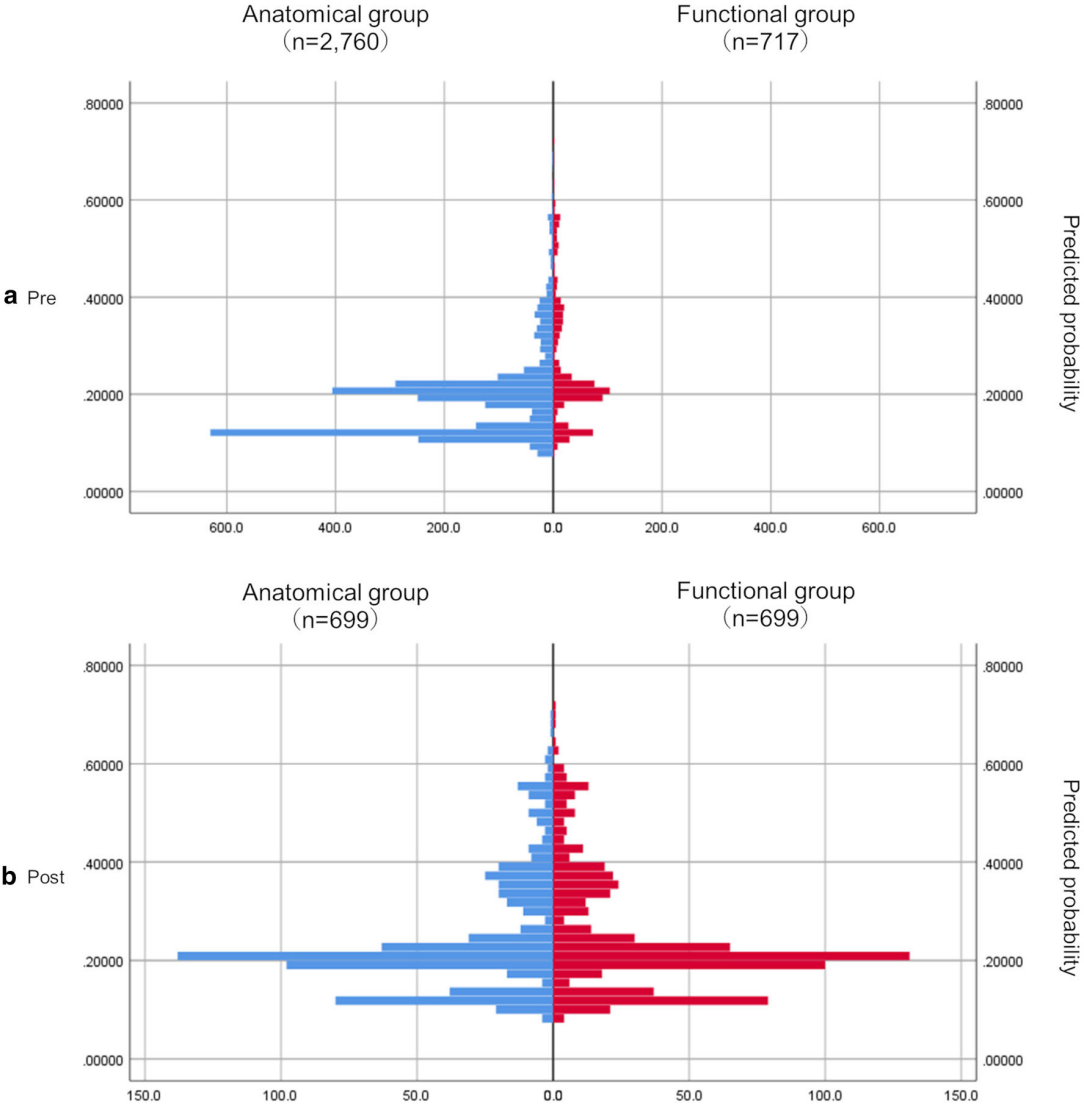
Score adjustment among the analysis groups with propensity score (PS) (comparison before and after matching in the overall patients [Category A]). We applied a multiple logistic regression analysis model

Regarding the bias related to patient background, it was reduced as much as possible using the propensity score (PS). In order to predict the dependent factors with which the value with functional ischemia evaluation is 1 and 0 for no evaluation, appropriate explanatory variables (covariates) were selected by the backward stepwise method in the multivariate logistic regression model. Explanatory variables were selected from sex, age, comorbidity, medication, and other factors (the factors are shown as Table S2). PS was performed for each of the three categories, and in category A, age, vasodilators, antihyperlipidemic, inotropic, antiarrhythmic, diuretic, and CKD were used as explanatory variables (Table S3). Furthermore, the PS for each case was calculated, the number of samples is aligned by applying the 1:1 matching method (Greedy matching), and the data distribution and balance of both groups were confirmed (examination of the summary statistics for each group) (Figure 2). Similar calculations were performed for categories B and C (Figure S1 and Figure S2).

### Study Parameters

In this study, we performed the observation for 36 months using the index day as the starting point for each modality combination in the study arms with matched patient backgrounds. The study was conducted from a social viewpoint (standpoint of public insurers). The primary endpoint was CEA, and the secondary endpoints were major adverse cardiovascular events (MACE) or life years (LYs unit: year) and medical costs. CEA was the ratio of the related cardiac medical costs for 36 months to LYs (US$/LY). MACE referred to cardiac death, ACS events, and hospitalization due to cardiac failure. The broadly defined defer rate was considered to indicate OMT being selected (rate of cardiac revascularization not being performed) was organized in a period of 3 months, considering the relevance with testing and treatment. Coronary revascularization also included scheduled PCI from the viewpoint of medical economics-based evaluation. The index used for costs was the amount paid to medical facilities under the national health insurance system. Indirect medical costs (patient travel costs and so on) were excluded from our analysis because we only included the scope of costs directly calculated as public medical costs. Costs were calculated to include initial examination/repeat examination, guidance, testing/diagnosis, imaging/interpretation, prescription/medication, administration/injection, procedure/surgery, and recuperation/rehabilitation. The points used in medical service bills were converted as 1 point = 10 yen. Japanese yen were converted to US dollars based on the mean conversion rate from 2014 to 2018 (1 US$ = 105.1 yen).

We used T-testing to examine mean population differences in this study. We used the chi-squared test to compare patient backgrounds and test for independence in the relationship between costs and effects. Survival curves were drawn using the Kaplan–Meier method, and log-rank testing was performed. The statistical analysis software used was SPSS version 26.0 (IBM Corp., Armonk, New York). The level of statistical significance was set at 5%, and mean values were expressed as standard deviations.

## Results

### Analysis of Subject Backgrounds

This study targeted a total of 3,477 subjects. PS was used to match patient backgrounds by category for the anatomical and functional group combinations.

For Category A (n = 1,398), which consisted of all cases, the male patients’ ratio was 68.1% in the anatomical group vs 71.7% in the functional group (*P* = .145), and ages were 55.01 ± 8.63 vs 54.60 ± 8.63 years, respectively (*P* = .374). The main diseases at baseline were hypertension in 58.4% vs 62.8%, respectively, (*P* = .090), dyslipidemia in 57.5% vs 57.2%, respectively (*P* = .914), and diabetes in 43.8% vs 42.5% of patients, respectively (*P* = .627). Patients’ pharmacotherapy history revealed that vasodilators were used by 50.5% vs 51.9%, respectively (*P* = .593), hypotensive agents by 41.6% vs 40.3%, respectively (*P* = .625), antihyperlipidemic agents by 29.6% vs 27.8%, respectively (*P* = .442), HMG-CoA reductase inhibitors (statins) by 27.5% vs 25.0%, respectively (*P* = .301), and antiarrhythmic agents by 2.6% vs 5.3% of patients, respectively (*P* = .009; Table 1).

**Table 1:** Background characteristics of the patients in the functional group and the anatomical group (by category)

	A: Over all			B: Non-invasive examination			C: Invasive examination		
	Anatomical group (n = 699)	Functional group (n = 699)	*P* value	CTA group (n = 402)	SPECT group (n = 402)	*P* value	CAG group (n = 261)	FFR/SPECT group (n = 261)	*P* value
Male, n (%)	476 (68.1)	501 (71.7)	.145	251 (62.4)	266 (66.2)	.270	209 (80.1)	209 (80.1)	1.000
Mean age ± SD (years)	55.01 ± 8.63	54.60 ± 8.63	.374	53.29 ± 8.36	53.93 ± 9.02	.299	55.05 ± 9.02	55.38 ± 7.92	.658
Comorbidity									
Hypertension, n (%)	408 (58.4)	439 (62.8)	.090	240 (59.7)	235 (58.5)	.720	179 (68.6)	184 (70.5)	.634
Dyslipidemia, n (%)	402 (57.5)	400 (57.2)	.914	225 (56.0)	213 (53.0)	.395	180 (69.0)	166 (63.6)	.195
Diabetes mellitus, n (%)	306 (43.8)	297 (42.5)	.627	163 (40.5)	159 (39.6)	.773	125 (47.9)	118 (45.2)	.539
CKD, n (%)	115 (16.5)	122 (17.5)	.618	63 (15.7)	65 (16.2)	.847	40 (15.3)	41 (15.7)	.904
Cerebrovascular disease, n (%)	85 (12.2)	80 (11.4)	.679	45 (11.2)	43 (10.7)	.821	41 (15.7)	30 (11.5)	.160
PAD, n (%)	43 (6.2)	30 (4.3)	.118	23 (5.7)	16 (4.0)	.251	14 (5.4)	13 (5.0)	.843
Medication									
Vasodilator, n (%)	353 (50.5)	363 (51.9)	.593	169 (42.0)	172 (42.8)	.830	176 (67.4)	176 (67.4)	1.000
Hypotensive, n (%)	291 (41.6)	282 (40.3)	.625	162 (40.3)	151 (37.6)	.426	109 (41.8)	109 (41.8)	1.000
Antihyperlipidemic, n (%)	207 (29.6)	194 (27.8)	.442	100 (24.9)	101 (25.1)	.935	84 (32.2)	77 (29.5)	.507
Statin, n (%)	192 (27.5)	175 (25.0)	.301	85 (21.1)	88 (21.9)	.797	81 (31.0)	73 (28.0)	.443
Antiplatelet, n (%)	172 (24.6)	164 (23.5)	.617	64 (15.9)	59 (14.7)	.624	93 (35.6)	93 (35.6)	1.000
Diuretic, n (%)	120 (17.2)	139 (19.9)	.191	56 (13.9)	60 (14.9)	.688	52 (19.9)	61 (23.4)	.339
Antidiabetic, n (%)	84 (12.0)	82 (11.7)	.869	37 (9.2)	39 (9.7)	.809	38 (14.6)	35 (13.4)	.705
Inotropic, n (%)	35 (5.0)	47 (6.7)	.172	19 (4.7)	20 (5.0)	.870	9 (3.4)	19 (7.3)	.052
Anticoagulant, n (%)	33 (4.7)	41 (5.9)	.339	20 (5.0)	26 (6.5)	.362	8 (3.1)	13 (5.0)	.265
Antiarrhythmic, n (%)	18 (2.6)	37 (5.3)	.009	20 (5.0)	25 (6.2)	.443	15 (5.7)	12 (4.6)	.553
Others									
Chest pain, n (%)	68 (9.7)	62 (8.9)	.581	58 (14.4)	37 (9.2)	.022	17 (6.5)	22 (8.4)	.405
Dialysis, n (%)	26 (3.7)	35 (5.0)	.239	13 (3.2)	14 (3.5)	.845	13 (5.0)	14 (5.4)	.843

*CKD*, chronic kidney disease; *PAD*, peripheral arterial disease; *Statin*, HMG-CoA reductase inhibitor; *CTA*, coronary computed tomography angiography; *SPECT*, cardiac single-photon emission computed tomography; *CAG*, coronary angiography; *FFR*, coronary fractional flow reserve; *SD*, standard deviation

For Category B (n = 804), which included combinations of non-invasive modalities, the male patients’ ratio was 62.4% in the CTA group vs 66.2% in the SPECT group (*P* = .270), ages were 53.29 ± 8.36 vs 53.93 ± 9.02 years, respectively (*P* = .299), and chest pain was present in 14.4% vs 9.2% of patients, respectively (*P* = .022). No major differences were noted between the groups in terms of disease at baseline or history of pharmacotherapy.

For Category C (n = 522), which included combinations of invasive modalities, the male patients’ ratio was 80.1% in the CAG group vs 80.1% in the FFR/SPECT group (*P* = 1.000), ages were 55.05 ± 9.02 vs 55.38 ± 7.92 years, respectively (*P* = .658), and inotropic agents was present in 3.4% vs 7.3% of patients, respectively (*P* = .052). No major differences were noted between the groups in terms of disease at baseline or history of pharmacotherapy.

### Evaluation of Clinical Effects

The defer rate (rate of not undergoing coronary revascularization within 3 months) for Category A, consisting of overall subjects, was 93.0% and 92.3% in the functional and anatomical groups, respectively (Figure 1). For Category B, it was 98.3% and 94.8% in the SPECT and CTA groups, respectively. For Category C, it was 84.3% and 77.8% in the FFR/SPECT and CAG groups, respectively.

**Figure 3:**
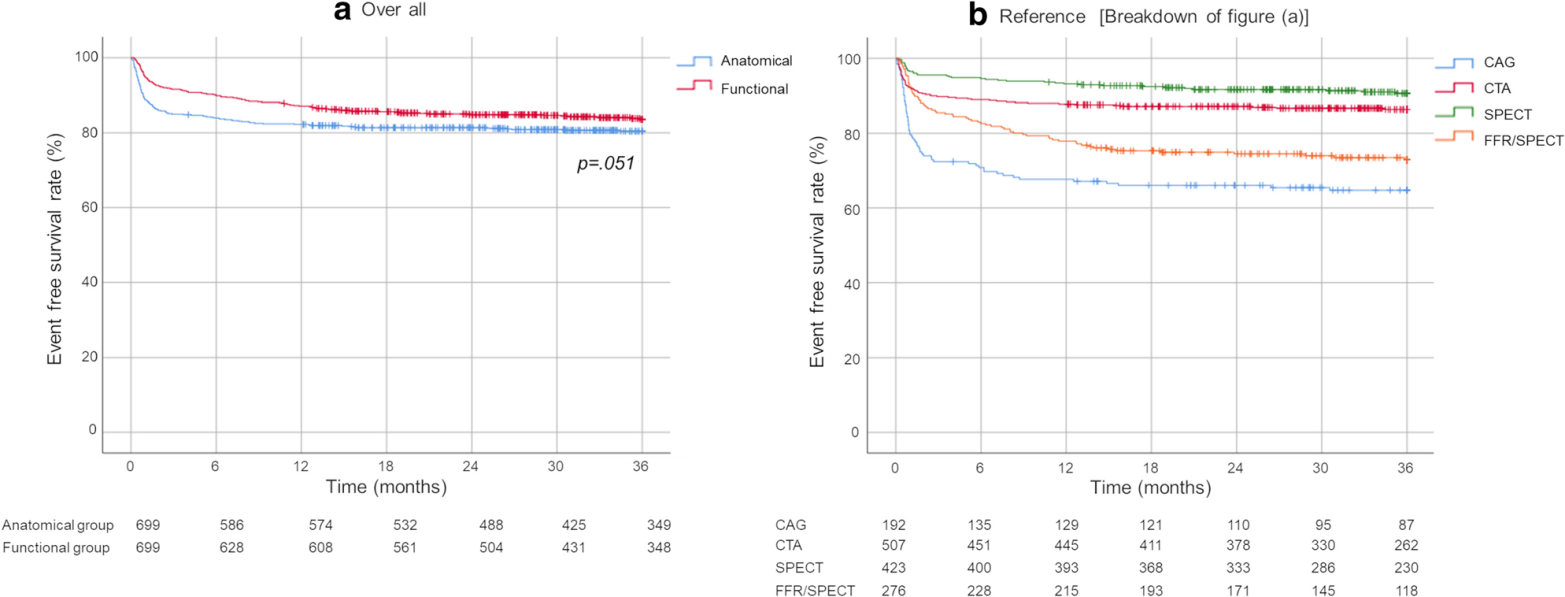
Comparison of the MACE onset rates between the functional and anatomical groups in the overall patients (Category A). Survival curves were drawn using the Kaplan–Meier method, and log-rank testing was performed

**Figure 4:**
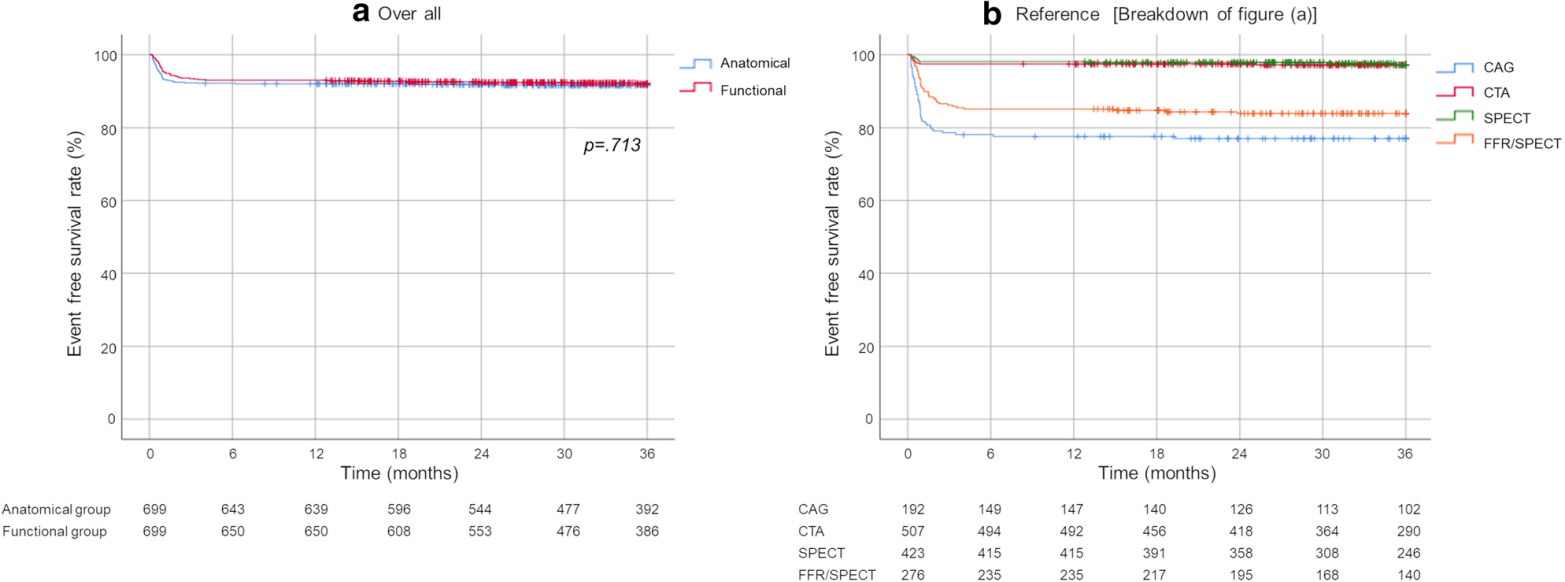
Comparison of the coronary revascularization rates between the functional and anatomical groups in the overall patients (Category A)

MACE evaluation, which was performed using survival curves and the log-rank test, revealed no statistically significant differences between the functional and anatomical groups in Category A at 36 months (*P* = .051, Figure 3 [a]). Analysis of coronary revascularization frequency using survival curves also revealed no statistically significant differences between the functional and anatomical groups in Category A at 36 months (*P* = .713, Figure 4 [a]). The emergency coronary revascularization (including ACS) rates were 1.43% and 1.00% in the anatomical (< 3 months: 1.14%, ≥ 3 months: 0.29%) and functional (< 3 months: 0.43%, ≥ 3 months: 0.57%) groups, respectively.

**Figure 5:**
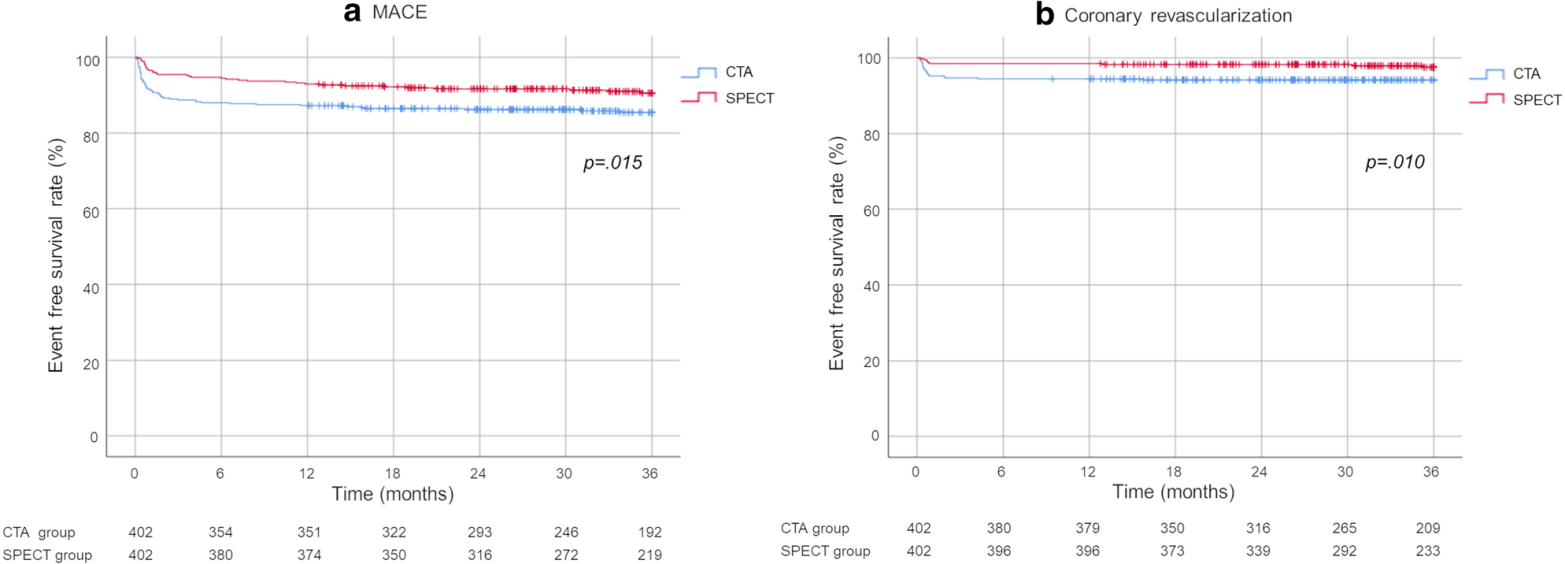
Comparison of the MACE onset and coronary revascularization rates between the functional and anatomical groups for non-invasive testing (Category B)

For Category B, MACE onset and coronary revascularization implementation rates were both statistically significantly lower in the SPECT group than in the CTA group at 36 months (*P* = .015 and *P* = .010, respectively, Figure 5).

**Figure 6:**
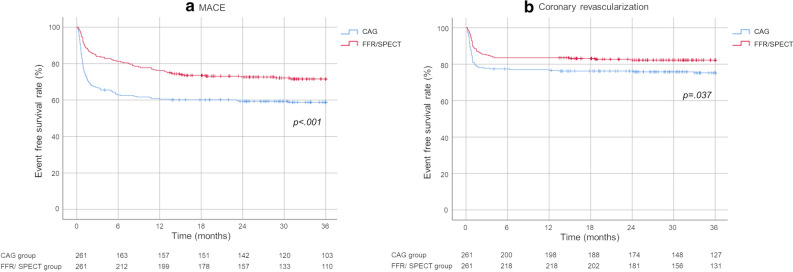
Comparison of the MACE onset and coronary revascularization rates between the functional and anatomical groups for invasive testing (Category C)

For Category C, MACE onset and coronary revascularization implementation rates were both statistically significantly lower in the FFR/SPECT group than in the CAG group at 36 months (*P* < .001, *P* = .037, respectively; Figure 6).

For Category A, LYs during the follow-up period from 12 to 36 months were slightly longer for the functional group than for the anatomical group, but no statistically significant difference was noted (2.666 ± 0.543 vs 2.663 ± 0.560 years, respectively, *P* = .916, Table 2).

**Table 2:** Comparison of the medical costs, LYs, and CEA in the functional group and the anatomical group (by category)

	A: Over all			B: Non-invasive examination			C: Invasive examination		
	Anatomical group (n = 699)	Functional group (n = 699)	*P* value	CTA group (n = 402)	SPECT group (n = 402)	*P* value	CAG group (n = 261)	FFR/SPECT group (n = 261)	*P* value
	Mean ± SD	Mean ± SD		Mean ± SD	Mean ± SD		Mean ± SD	Mean ± SD	
Life years (LYs, years)	2.663 ± 0.560	2.666 ± 0.543	.916	2.619 ± 0.562	2.675 ± 0.525	.146	2.695 ± 0.544	2.652 ± 0.561	.383
Total medical costs (US$)	7,038 ± 11,397	6,248 ± 8,653	.144	5,149 ± 8,535	4,059 ± 5,957	.036	13,587 ± 16,371	9,485 ± 11,190	.001
Hospitalization costs (US$)	4,430 ± 10,644	3,105 ± 7,588	.007	2,592 ± 7,791	1,197 ± 4,964	.003	10,350 ± 16,157	6,228 ± 10,024	.001
[Details]									
Medical care (US$)	2,141 ± 5,768	1,705 ± 4,472	.115	961 ± 3,103	602 ± 2,723	.082	6,003 ± 9,387	3,488 ± 6,057	< .001
Medication (US$)	287 ± 1,186	339 ± 883	.356	130 ± 1,164	112 ± 495	.777	833 ± 1,935	688 ± 1,241	.309
Special treatment materials (US$)	2,002 ± 5,392	1,061 ± 3,897	< .001	1,501 ± 4,648	483 ± 2,354	< .001	3,514 ± 6,648	2,052 ± 5,550	.007
Outpatient costs (US$)	2,608 ± 2,905	3,143 ± 3,205	.001	2,557 ± 2,508	2,862 ± 2,842	.107	3,237 ± 3,319	3,257 ± 3,310	.946
[Details]									
Medical care (US$)	1,080 ± 817	1,290 ± 946	< .001	1,086 ± 794	1,252 ± 846	.004	1,143 ± 859	1,229 ± 866	.254
Medication (US$)	1,528 ± 2,418	1,853 ± 2,659	.017	1,471 ± 2,073	1,610 ± 2,371	.376	2,094 ± 2,839	2,028 ± 2,856	.789
CEA (US$/LY)	2,902 ± 5,115	2,431 ± 3,433	.043	2,120 ± 3,750	1,551 ± 2,188	.009	5,404 ± 7,183	3,701 ± 4,511	.001

*CTA*, coronary computed tomography angiography; *SPECT*, cardiac single-photon emission computed tomography; *CAG*, coronary angiography; *FFR*, coronary fractional flow reserve; *SD*, standard deviation; *CEA*, cost-effective analysis

For Category B, LYs were longer for the SPECT group than for the CTA group, but no statistically significant difference was noted (2.675 ± 0.525 vs 2.619 ± 0.562 years, respectively, *P* = .146, Table 2).

Meanwhile, for Category C, although LYs were slightly shorter for the FFR/SPECT group than for the CAG group, no statistically significant difference was noted (2.652 ± 0.561 vs 2.695 ± 0.544 years, *P* = .383, Table 2).

### Analysis of Medical Costs

Analysis revealed that, for subjects overall (Category A), cumulative (total) medical costs were lower for the functional group than for the anatomical group, although no statistically significant difference was noted (6,248 ± 8,653 US$ vs 7,038 ± 11,397 US$, respectively, *P* = .144, Table 2). When medical costs were broken down, overall hospitalization costs were lower for the functional group than for the anatomical group, with a statistically significant difference noted (3,105 ± 7,588 US$ vs 4,430 ± 10,644 US$, respectively, *P* = .007, Table 2). We noted that although no major differences were observed for medical care costs and medication costs in hospitalized treatment, costs for special treatment materials were lower for the functional group than for the anatomical group, with a statistically significant difference noted (1,061 ± 3,897 US$ vs 2,002 ± 5,392 US$, respectively, *P* < .001). For PCI in particular, the mean number of coronary stents placed was significantly higher for the anatomical group than for the functional group (1.91 stents/case vs 1.17 stents/case, respectively, *P* = .001, Table 3). Meanwhile, outpatient medical costs were significantly higher for the functional group than for the anatomical group (3,143 ± 3,205 US$ vs 2,608 ± 2,905 US$, respectively, *P* = .001, Table 2).

**Table 3:** Comparison of the number of stents placed during PCI in the functional group and the anatomical group (by category)

Group	Number of patients who underwent coronary stent implantation	Average number of stent ± SD	*P* value
A: Over all			
Anatomical group	47	1.91 ± 1.35	.001
Functional group	36	1.17 ± 0.45	
B: Non-invasive examination			
CTA group	21	1.52 ± 0.93	.689
SPECT group	8	1.38 ± 0.74	
C: Invasive examination			
CAG group	41	1.71 ± 1.19	.003
FFR/SPECT group	28	1.11 ± 0.32	

*CTA*, coronary computed tomography angiography; *SPECT*, cardiac single-photon emission computed tomography; *CAG*, coronary angiography; *FFR*, coronary fractional flow reserve; *SD*, standard deviation; *CEA*, cost-effective analysis; *PCI*, percutaneous coronary intervention

For Category B, medical costs were significantly lower for the SPECT group than for the CTA group (4,059 ± 5,957 US$ vs 5,149 ± 8,535 US$, respectively *P* = .036, Table 2). Similar trends to those noted in Category A were also observed for hospitalization and outpatient treatment costs.

For Category C, medical costs were significantly lower for the FFR/SPECT group than for the CAG group (9,485 ± 11,190 US$ vs 13,587 ± 16,371 US$, respectively, *P* = .001, Table 2). Similar trends to those noted in Categories A and B were also noted for hospitalization costs.

### Cost-Effectiveness Evaluation

We performed CEA (annual medical costs per LYs) on the entire cohort of patients (Category A) to investigate the cost-effectiveness of the functional group compared to that of the anatomical group. We found that the CEA of the functional group was significantly better than the anatomical group (2,431 ± 3,433 US$/LY vs 2,902 ± 5,115 US$/LY, respectively, *P* = .043, Table 2).

Similarly, for Category B, the CEA was significantly better for the SPECT group than for the CTA group (1,551 ± 2,188 US$/LY vs 2,120 ± 3,750 US$/LY, respectively, *P* = .009, Table 2). Of the three categories, the most favorable trend was noted for CEA in Category B.

For Category C, the CEA was significantly better for the FFR/SPECT group than the CAG group (3,701 ± 4,511 US$/LY vs 5,404 ± 7,183 US$/LY, respectively, *P* = .001, Table 2).

## Discussion

### Summary of this Study

This study applied real-world data to evaluate medical economics of advanced diagnostic modalities for patients with stable CAD under long-term observation applying propensity score matching.

The results clearly indicated that the functional group had overall superior MACE and CEA results compared to those of the anatomical group (Category A). When non-invasive diagnostic tests were considered (Category B), the functional group (SPECT group) had more favorable results on MACE, coronary revascularization, total medical costs, hospitalization costs, and CEA compared to the anatomical group (CTA group). Likewise, when invasive diagnostic approaches were considered (Category C), the functional group (FFR/SPECT group) had more favorable results on MACE, coronary revascularization, total medical costs, hospitalization costs, and CEA compared to the anatomical group (CAG group).

Thus, the functional ischemia evaluation offers superior overall cost-effectiveness together with better outcomes when diagnostic and subsequent therapeutic decisions are made in patients with stable CAD. Particularly, when functional ischemia is non-invasively evaluated in patients undergoing PCI at a relatively early stage, which means the SPECT group in Category B, the best results for cost-effectiveness can be possibly achieved.

### Coronary Revascularization

For subjects in Category A, the long-term results after 36 months for MACE and coronary revascularization were significantly lower for CAG (including some CTA cases) than for the other testing modalities. In particular, coronary revascularization (including scheduled PCI) tended to be performed during the first 2 months after the index day when anatomical diagnostic approaches were used. This is probably because morphology-based decision-making was more likely to induce anatomical treatment using coronary revascularization when compared to the functional strategy. This is supported by the following findings: discordance is observed between coronary artery narrowing visually assessed and inducible ischemia assessed by FFR in the non-negligible number of patients with stable CAD,^22^,^23^ and the functional assessment reduced unnecessary (i.e., prognostically non-beneficial) coronary intervention when compared to the anatomical assessment alone.^4^,^5^

Despite its invasive nature, FFR has been widely used to identify functional ischemia and eligibility for elective PCI, particularly when coronary narrowing is equivocal or ≥ 50%. In retrospective studies, FFR leads to a higher probability of detecting multiple advanced coronary artery lesions compared to no FFR assessment. Consequently, we believe that selection bias could have been present for the FFR assessment, resulting in the higher rate of PCI for cardiovascular events. In the present study, therefore, the patient backgrounds in Category C, consisting of patients undergoing invasive diagnostic testing, were matched between the CAG and FFR/SPECT groups for pre-testing ECG and post-testing antiplatelet agent prescription in each modality. This determined the pre-test probability of cardiac catheter testing. These analyses clearly found that the FFR/SPECT group less frequently underwent PCI than did the CAG group, as shown previously.^5^,^6^,^9^

### Defer Rate and MACE

The defer rate was relatively high in this study. This trend was more evident in the non-invasive group, Category B, than in the invasive group, Category C. Due to invasive tests, Category C may have been more likely to subsequently derive invasive treatment. We interpreted this to be generally consistent with the situation in clinical settings. Similar to the aforementioned coronary revascularization result, the relatively high rate of MACE in the CAG group may have been affected by the limited diagnostic precision due to a lack of functional data on ischemia.^5^,^9^,^24^

The MACE observed in this study included a relatively high number of hospitalizations for arrhythmia. A major underlying disease requiring hospitalization treatment in Japan is heart failure in which CAD is a leading cause.^25^–^27^ We noted a similar trend in the present study. Considering the patients’ backgrounds, the risk factors for arrhythmia were basically identical in the CAG and FFR/SPECT groups. Cardiotonic agent use was less frequent in the CAG group and medical costs of outpatient treatment were relatively higher in the FFR/SPECT group when compared to each counterpart. These findings suggest that the FFR/SPECT group had fewer hospitalization events owing to the appropriate evaluation of functional myocardial ischemia and clinical risks and to risk-based treatment strategy, including OMT.

### Health Economic Evaluation

The medical cost data used included the medical service bills obtained under Japan’s public health insurance system. Given that the national public health insurance system is based on the assumption of all-inclusive national healthcare, almost all patients receiving stable CAD-related treatment and the details of such treatment were officially covered. The medical big data (*TheBD*), the data source used in this study, accounted for approximately 7% of all medical service bill data in Japan and were mainly obtained from corporate health insurance societies. The case composition and regional distribution of our data suggested that the present results were generally representative of the current clinical state of CAD management in Japan. The scope of calculated medical costs included all treatments related to stable CAD. The following costs, however, were excluded from the analysis: indirect medical costs, such as non-publicly provided meal and travel costs unrelated to treatment, as well as costs related to elderly long-term care. Although the data analyzed in this study included cases with organic cardiac disease risks, it was a relatively young population (approximately 55 years old).

Concerning CEA results, such as medical costs during a follow-up period of 36 months and LYs (US$/LY), the results were significantly better for the functional group than for the anatomical group. A detailed breakdown of the medical costs revealed that hospitalization costs were markedly lower, whereas the outpatient costs were markedly higher for the functional group. It is noted that costs for special treatment materials for the functional group were approximately half that of the anatomical group. This is probably because the number of related hospitalization events and the number of stents placed during PCI were significantly lower in the functional group than in the anatomical group. In contrast, the relatively high outpatient costs for the functional group appeared to be due to the high proportion of OMT cases that could be controlled using outpatient pharmacotherapy. In short, total medical costs were relatively lower for the functional group. The results for MACE, an effects index, were better for the functional group than for the anatomical group, whereas the cost index of the total medical costs was lower. These findings suggest that the functional group had a “dominant” position in the cost-effectiveness plane of the incremental cost-effectiveness ratio.^28^

### Limitations and Prospects

This study had some limitations. First, the data on test values or medical interview results were not included, making it difficult to perform detailed analysis on patient backgrounds in relation to the actual clinical conditions. Second, because the data source selection conditions limited the sample size of elderly cases analyzed here, it was difficult to correlate conditions, such as heart failure with preserved ejection fraction, with PCI strategy and outcomes in the elderly population. Third, although its design meant that pseudo-allocation with PS processing was performed, the data source was not based on a randomized controlled study, suggesting the insufficient exclusion of selection bias.

Prospective clinical verification of the present findings is required in a future study. In particular, a more long-term clinical observation of PCI-deferred cases is needed to conclude an outcome analysis from both the clinical and economic viewpoints. PCI-deferral criteria based on ischemia-based and risk-based strategy are not established, and a long-term follow-up protocol with OMT strategy and appropriate reassessment of ischemia condition is required to be standardized. As shown by the very recent ISCHEMIA trial,^29^ it is also important to evaluate the health outcomes by using the Seattle Angina Questionnaire or Quality-Adjusted Life Year. The age-related differences, treatment result, and billing state databases for elderly patients need to be analyzed in the future for Japan’s aging society.

Due to global increases in the disease burden and the economic burden of the overall medical system, the establishment of a clinically appropriate and cost-effective system for stable CAD is one of the important health policy issues needing to be resolved. The present findings should be further discussed with the aim of constructing an economically sustainable medical system for stable CAD management. From the perspective of the overall optimization of the healthcare system, we expect that discussing the balance of clinical practice and economics will aid in the further advancement of clinical practice.^30^ Our results suggest that promoting the more widespread implementation of functional ischemia evaluation could greatly contribute to the sustainable development of a widely acceptable medical system.

In summary, the preset findings clearly demonstrated that the functional group had better clinical outcomes and lower cumulative medical costs compared to the anatomical group, improving cost-effectiveness modality composition for functional myocardial ischemia evaluations. In particular, a non-invasive diagnostic approach is superior in terms of medical economics and prognosis, indicating promising outpatient management strategy for stable CAD where the prevalence of coronary disease and heart failure with ischemic etiology have been increasing considerably.

## New Knowledge Gained

This study was a medical economics-based evaluation applying cost-effectiveness analysis (CEA) to investigate advanced testing and diagnosis for coronary revascularization in patients with stable coronary artery disease (CAD), including treatment options and prognosis, while focusing on functional ischemia evaluation. Our findings revealed that long-term clinical outcomes showed greater improvement and cumulative medical costs was lower in the functional group than in the anatomical group, suggesting that the modality composition of functional myocardial ischemia evaluation offers superior cost-effectiveness.

## Electronic supplementary material

Electronic supplementary material 1 (DOCX 41 kb)

Electronic supplementary material 2 (JPG 206 kb)

Electronic supplementary material 3 (JPG 203 kb)

Electronic supplementary material 4 (PPTX 3885 kb)

Electronic supplementary material 5 (MP3 1892 kb)

## Abbreviations

ACS: Acute coronary syndrome
CAD: Coronary artery disease
CAG: Coronary angiography
CEA: Cost-effectiveness analysis
CTA: Computed tomography angiography
FFR: Fractional flow reserve
LY: Life years
MACE: Major adverse cardiovascular events
OMT: Optimal medical therapy
PCI: Percutaneous coronary intervention
PS: Propensity score
SPECT: Single photon emission computed tomography

## References

[1] The Japanese registry of all cardiac and vascular diseases (Announced in FY2018). National Cerebral and Cardiovascular Center Hospital. 2019. http://www.j-circ.or.jp/jittai_chosa/jittai_chosa2016web.pdf. Accessed 1 April 2020.

[2] Takura T (2019). Current trends in medical economics in the circulatory field—socioeconomics background and research issue. Circ Rep.

[3] J-PCI Registry 2018 Report (FY2017 Aggregate Results). Japanese Association of Cardiovascular Intervention and Therapeutics. 2018. Available online: http://www.cvit.jp/files/registry/annual-report/j-pci/2018.pdf. Accessed 1 April 2020.

[4] Tonino PA, Fearon WF, De Bruyne B, Oldroyd KG, Leesar MA, Ver Lee PN (2010). Angiographic versus functional severity of coronary artery stenoses in the FAME study fractional flow reserve versus angiography in multivessel evaluation. J Am Coll Cardiol..

[5] De Bruyne B, Fearon WF, Pijls NH, Barbato E, Tonino P, Piroth Z (2014). Fractional flow reserve-guided PCI for stable coronary artery disease. N Engl J Med..

[6] Zimmermann FM, Ferrara A, Johnson NP, van Nunen LX, Escaned J, Albertsson P (2015). Deferral vs performance of percutaneous coronary intervention of functionally non-significant coronary stenosis: 15-year follow-up of the DEFER trial. Eur Heart J..

[7] Boden WE, O’Rourke RA, Teo KK, Hartigan PM, Maron DJ, Kostuk WJ (2007). Optimal medical therapy with or without PCI for stable coronary disease. N Engl J Med..

[8] Meijboom WB, Van Mieghem CA, van Pelt N, Weustink A, Pugliese F, Mollet NR (2008). Comprehensive assessment of coronary artery stenoses: computed tomography coronary angiography versus conventional coronary angiography and correlation with fractional flow reserve in patients with stable angina. J Am Coll Cardiol..

[9] Tonino PA, De Bruyne B, Pijls NH, Siebert U, Ikeno F, van’t Veer M (2009). Fractional flow reserve versus angiography for guiding percutaneous coronary intervention. N Engl J Med..

[10] Hachamovitch R, Rozanski A, Shaw LJ, Stone GW, Thomson LE, Friedman JD (2011). Impact of ischaemia and scar on the therapeutic benefit derived from myocardial revascularization vs medical therapy among patients undergoing stress-rest myocardial perfusion scintigraphy. Eur Heart J..

[11] Weintraub WS, Hartigan PM, Mancini GBJ, Teo KK, Maron DJ, Spertus JA (2019). Effect of coronary anatomy and myocardial ischemia on long-term survival in patients with stable ischemic heart disease. Circ Cardiovasc Qual Outcomes..

[12] Stone GW, Maehara A, Lansky AJ, de Bruyne B, Cristea E, Mintz GS (2011). A prospective natural history study of coronary atherosclerosis. N Engl J Med..

[13] Shaw LJ, Berman DS, Maron DJ, Mancini GB, Hayes SW, Hartigan PM (2008). Optimal medical therapy with or without percutaneous coronary intervention to reduce ischemic burden: results from the Clinical Outcomes Utilizing Revascularization and Aggressive Drug Evaluation (COURAGE) trial nuclear substudy. Circulation..

[14] Nanasato M, Matsumoto N, Nakajima K, Chikamori T, Moroi M, Takehana K (2018). Prognostic impact of reducing myocardial ischemia identified using ECG-gated myocardial perfusion SPECT in Japanese patients with coronary artery disease: J-ACCESS4 study. Int J Cardiol..

[15] Frye RL, August P, Brooks MM, Hardison RM, Kelsey SF, BARI 2D Study Group (2009). A randomized trial of therapies for type 2 diabetes and coronary artery disease. N Engl J Med..

[16] Fearon WF, Bornschein B, Tonino PA, Gothe RM, Bruyne BD, Pijls NH (2010). Economic evaluation of fractional flow reserve-guided percutaneous coronary intervention in patients with multivessel disease. Circulation..

[17] Pijls NH, van Schaardenburgh P, Manoharan G, Boersma E, Bech JW, van’t Veer M (2007). Percutaneous coronary intervention of functionally nonsignificant stenosis: 5-year follow-up of the DEFER Study. J Am Coll Cardiol..

[18] Kawase Y, Matsuo H, Akasaka T, Shiono Y, Tanaka N, Amano T (2019). Clinical use of physiological lesion assessment using pressure guidewires: an expert consensus document of the Japanese Association of Cardiovascular Intervention and Therapeutics. Cardiovasc Interv Ther..

[19] Takura T, Tachibana K, Isshiki T, Sumitsuji S, Kuroda T, Mizote I (2017). Preliminary report on a cost-utility analysis of revascularization by percutaneous coronary intervention for ischemic heart disease. Cardiovasc Interv Ther..

[20] Tanaka N, Kohsaka S, Murata T, Akasaka T, Kadota K, Uemura S (2019). Treatment strategy modification and its implication on the medical cost of fractional flow reserve-guided percutaneous coronary intervention in Japan. J Cardiol..

[21] Benchimol EI, Smeeth L, Guttmann A, Harron K, Moher D, Petersen I (2015). The REporting of studies Conducted using Observational Routinely-collected health Data (RECORD) Statement. PLoS Med..

[22] Nakamura M, Yamagishi M, Ueno T, Hara K, Ishiwata S, Itoh T (2014). Prevalence of visual-functional mismatch regarding coronary artery stenosis in the CVIT-DEFER registry. Cardiovasc Interv Ther..

[23] Nakamura M, Yamagishi M, Ueno T, Hara K, Ishiwata S, Itoh T (2015). Modification of treatment strategy after FFR measurement: CVIT-DEFER registry. Cardiovasc Interv Ther..

[24] Bech GJ, De Bruyne B, Pijls NH, de Muinck ED, Hoorntje JC, Escaned J (2001). Fractional flow reserve to determine the appropriateness of angioplasty in moderate coronary stenosis: a randomized trial. Circulation..

[25] Tsutsui H, Tsuchihashi-Makaya M, Kinugawa S, Goto D, Takeshita A, JCARE-CARD Investigators (2006). Clinical characteristics and outcome of hospitalized patients with heart failure in Japan. Circ J..

[26] Shiba N, Watanabe J, Shinozaki T, Koseki Y, Sakuma M, Kagaya Y, Shirato K, CHART Investigators (2004). Analysis of chronic heart failure registry in the Tohoku district: third year follow-up. Circ J..

[27] Shiba N, Nochioka K, Miura M, Kohno H, Shimokawa H, CHART-2 Investigators (2011). Trend of westernization of etiology and clinical characteristics of heart failure patients in Japan–first report from the CHART-2 study. Circ J..

[28] American College of Cardiology/American Heart Association Task Force. ACC/AHA statement on cost/value methodology in clinical practice guidelines and performance measures. 2014. 10.1016/j.jacc.2014.03.016 24681044

[29] Spertus JA, Jones PG, Maron DJ, O’Brien SM, Reynolds HR, Rosenberg Y (2020). Health-status outcomes with invasive or conservative care in coronary disease. N Engl J Med..

[30] Takura T (2018). An evaluation of clinical economics and cases of cost-effectiveness. Intern Med..

