# Supplementary material for: Health economics-based verification of functional myocardial ischemia evaluation of stable coronary artery disease in Japan: A long-term longitudinal study using propensity score matching
Source: J Nucl Cardiol. 2021 Jan 18;29(3):1356–69. doi: 10.1007/s12350-020-02502-9 (PMC9162976; doi:10.1007/s12350-020-02502-9)
Supplement: Supplementary file 1 — Electronic supplementary material 1 (DOCX 41 kb) [file 12350_2020_2502_MOESM1_ESM.docx]

**Table S1.** Database overview (*TheBD*)

1. Unit: Notation is "a total of roughly 1,000 people" (patient sample).
2. Conditions: In the aggregation, the results were narrowed down under the following conditions.

　・Aggregation period is 2012-2019

　・Excludes single samples not used for longitudinal studies

　・Exclude dental single samples

| **Male-female ratio (overall)** | |  |
| --- | --- | --- |
| **Sex** | **Total number of patients** | **Ratio** |
| **Total** | **7,071** | **100.0%** |
| Male | 3,337 | 47.2% |
| Female | 3,733 | 52.8% |
| **Service type (medical, dispensing, dentistry)** | | |
| **Receipt type** | **Total number of patients** | **Ratio** |
| **Total** | **7,071** | **100.0%** |
| Medical | 6,816 | 96.4% |
| Dentistry | 4,221 | 59.7% |
| Dispensing | 6,201 | 87.7% |
| **Inpatient/Outpatient ratio (medical)** | |  |
| **Inpatient/Outpatient** | **Total number of patients** | **Ratio** |
| **Total** | **7,071** | **100.0%** |
| Inpatient | 1,046 | 14.8% |
| Outpatient | 7,064 | 99.9% |

**Table S2.** Background characteristics of the patients in the functional group and the anatomical group (by category) before matching

|  | **A: Over all** | | | **B: Non-invasive examination** | | | **C: Invasive examination** | | |
| --- | --- | --- | --- | --- | --- | --- | --- | --- | --- |
|  | Anatomical group  (n=2,760) | Functional group  (n=717) | *p-*value | CTA group  (n=2,115) | SPECT group  (n=436) | *p-*value | CAG group  (n=645) | FFR/SPECT group  (n=281) | *p-*value |
| Male, n (%) | 1856 (67.2) | 516 (72.0) | 0.016 | 1337 (63.2) | 293 (67.2) | 0.115 | 519 (80.5) | 223 (79.4) | 0.698 |
| Mean age ± SD (years) | 53.75 ± 8.40 | 54.56 ± 8.57 | 0.024 | 53.21 ± 8.21 | 54.06 ± 8.85 | 0.050 | 55.55 ± 8.77 | 55.32 ± 8.09 | 0.709 |
| Comorbidity |  |  |  |  |  |  |  |  |  |
| Hypertension, n (%) | 1577 (57.1) | 453 (63.2) | 0.003 | 1123 (53.1) | 257 (58.9) | 0.026 | 454 (70.4) | 196 (69.8) | 0.846 |
| Dyslipidemia, n (%) | 1482 (53.7) | 413 (57.6) | 0.061 | 1042 (49.3) | 235 (53.9) | 0.078 | 440 (68.2) | 178 (63.3) | 0.148 |
| Diabetes mellitus, n (%) | 1048 (38.0) | 313 (43.7) | 0.005 | 735 (34.8) | 185 (42.4) | 0.002 | 313 (48.5) | 128 (45.6) | 0.405 |
| CKD, n (%) | 210 (7.6) | 136 (19.0) | < 0.001 | 120 (5.7) | 92 (21.1) | < 0.001 | 90 (14.0) | 44 (15.7) | 0.498 |
| Cerebrovascular  disease, n (%) | 280 (10.1) | 84 (11.7) | 0.221 | 174 (8.2) | 50 (11.5) | 0.029 | 106 (16.4) | 34 (12.1) | 0.090 |
| PAD, n (%) | 161 (5.8) | 34 (4.7) | 0.258 | 114 (5.4) | 20 (4.6) | 0.494 | 47 (7.3) | 14 (5.0) | 0.194 |
| Medication |  |  |  |  |  |  |  |  |  |
| Vasodilator, n (%) | 1680 (60.9) | 369 (51.5) | < 0.001 | 1123 (53.1) | 193 (44.3) | 0.001 | 557 (86.4) | 176 (62.6) | < 0.001 |
| Hypotensive, n (%) | 971 (35.2) | 295 (41.1) | 0.003 | 683 (32.3) | 176 (40.4) | 0.001 | 228 (44.7) | 119 (42.3) | 0.516 |
| Antihyperlipidemic, n (%) | 504 (18.3) | 206 (28.7) | < 0.001 | 242 (11.4) | 126 (28.9) | < 0.001 | 262 (40.6) | 80 (28.5) | < 0.001 |
| Statin, n (%) | 473 (17.1) | 185 (25.8) | < 0.001 | 218 (10.3) | 109 (25.0) | < 0.001 | 255 (39.5) | 76 (27.0) | < 0.001 |
| Antiplatelet, n (%) | 509 (18.4) | 174 (24.3) | < 0.001 | 135 (6.4) | 78 (17.9) | < 0.001 | 374 (58.0) | 96 (34.2) | < 0.001 |
| Diuretic, n (%) | 246 (8.9) | 154 (21.5) | < 0.001 | 108 (5.1) | 86 (19.7) | < 0.001 | 138 (21.4) | 68 (24.2) | 0.346 |
| Antidiabetic, n (%) | 182 (6.6) | 93 (13.0) | < 0.001 | 69 (3.3) | 55 (12.6) | < 0.001 | 113 (17.5) | 38 (13.5) | 0.130 |
| Inotropic, n (%) | 78 (2.8) | 49 (6.8) | < 0.001 | 45 (2.1) | 27 (6.2) | < 0.001 | 33 (5.1) | 22 (7.8) | 0.108 |
| Anticoagulant, n (%) | 101 (3.7) | 42 (5.9) | 0.008 | 76 (3.6) | 29 (6.7) | 0.003 | 25 (3.9) | 13 (4.6) | 0.597 |
| Antiarrhythmic, n (%) | 154 (5.6) | 37 (5.2) | 0.661 | 120 (5.7) | 25 (5.7) | 0.961 | 34 (5.3) | 12 (4.3) | 0.519 |
| Others |  |  |  |  |  |  |  |  |  |
| Chest pain, n (%) | 319 (11.6) | 63 (8.8) | 0.034 | 274 (13.0) | 38 (8.7) | 0.014 | 45 (7.0) | 25 (8.9) | 0.310 |
| Dialysis, n (%) | 44 (1.6) | 40 (5.6) | < 0.001 | 13 (0.6) | 24 (5.5) | < 0.001 | 31 (4.8) | 16 (5.7) | 0.572 |

CKD, chronic kidney disease; PAD, peripheral arterial disease; Statin, HMG-CoA reductase inhibitor; CTA, coronary computed tomography angiography; SPECT, cardiac single photon emission computed tomography; CAG, coronary angiography; FFR, coronary fractional flow reserve; SD, standard deviation

**Table S3.** Detailed breakdown of the covariance adjustment for propensity score (PS)

|  | Predictors | odds ratio | 95% confidence interval | | *p-*value |
| --- | --- | --- | --- | --- | --- |
|  |  |  | Lower limit | Upper limit |  |
| **A: Over all** | |  |  |  |  |
|  | Age | 1.012 | 1.001 | 1.022 | 0.027 |
|  | Vasodilators | 0.515 | 0.430 | 0.616 | < 0.001 |
|  | Antihyperlipidemic | 1.739 | 1.413 | 2.140 | < 0.001 |
|  | Inotropic | 1.686 | 1.119 | 2.542 | 0.013 |
|  | Antiarrhythmic | 2.226 | 1.723 | 2.877 | < 0.001 |
|  | Diuretic | 0.664 | 0.450 | 0.980 | 0.039 |
|  | CKD | 2.390 | 1.866 | 3.061 | < 0.001 |
| **B: Non-invasive examination** | | |  |  |  |
|  | Vasodilators | 0.473 | 0.375 | 0.597 | < 0.001 |
|  | Antihyperlipidemic | 2.386 | 1.744 | 3.263 | < 0.001 |
|  | Antiplatelet | 1.849 | 1.263 | 2.705 | 0.002 |
|  | Inotropic | 2.117 | 1.194 | 3.753 | 0.010 |
|  | Diuretic | 2.852 | 1.981 | 4.106 | < 0.001 |
|  | Antiarrhythmic | 0.545 | 0.330 | 0.899 | 0.017 |
|  | CKD | 2.805 | 1.983 | 3.968 | < 0.001 |
|  | Dialysis | 3.022 | 1.360 | 6.716 | 0.007 |
| **C: Invasive examination** | |  |  |  |  |
|  | Vasodilators | 0.300 | 0.213 | 0.423 | < 0.001 |
|  | Hypotensive | 1.593 | 1.144 | 2.218 | 0.006 |
|  | Antiplatelet | 0.385 | 0.276 | 0.536 | < 0.001 |

CKD, chronic kidney disease

Dependent variable; anatomical examination = 0, functional ischemic examination = 1
