# Supplementary figures and images for: Health economics-based verification of functional myocardial ischemia evaluation of stable coronary artery disease in Japan: A long-term longitudinal study using propensity score matching
Source: J Nucl Cardiol. 2021 Jan 18;29(3):1356–69. doi: 10.1007/s12350-020-02502-9 (PMC9162976; doi:10.1007/s12350-020-02502-9)

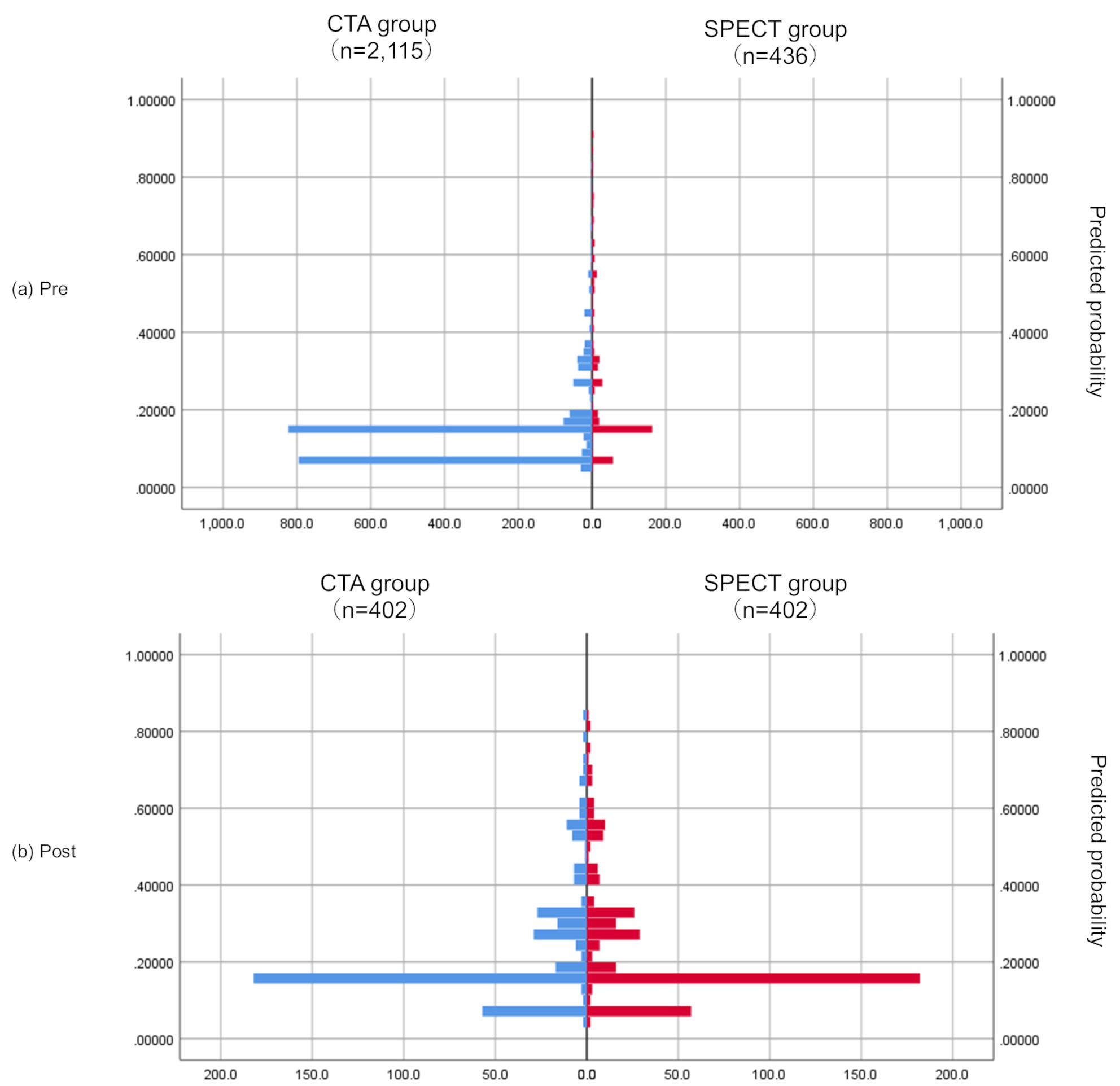

Supplement: Supplementary file 2 — Electronic supplementary material 2 (JPG 206 kb) [file 12350_2020_2502_MOESM2_ESM.jpg]

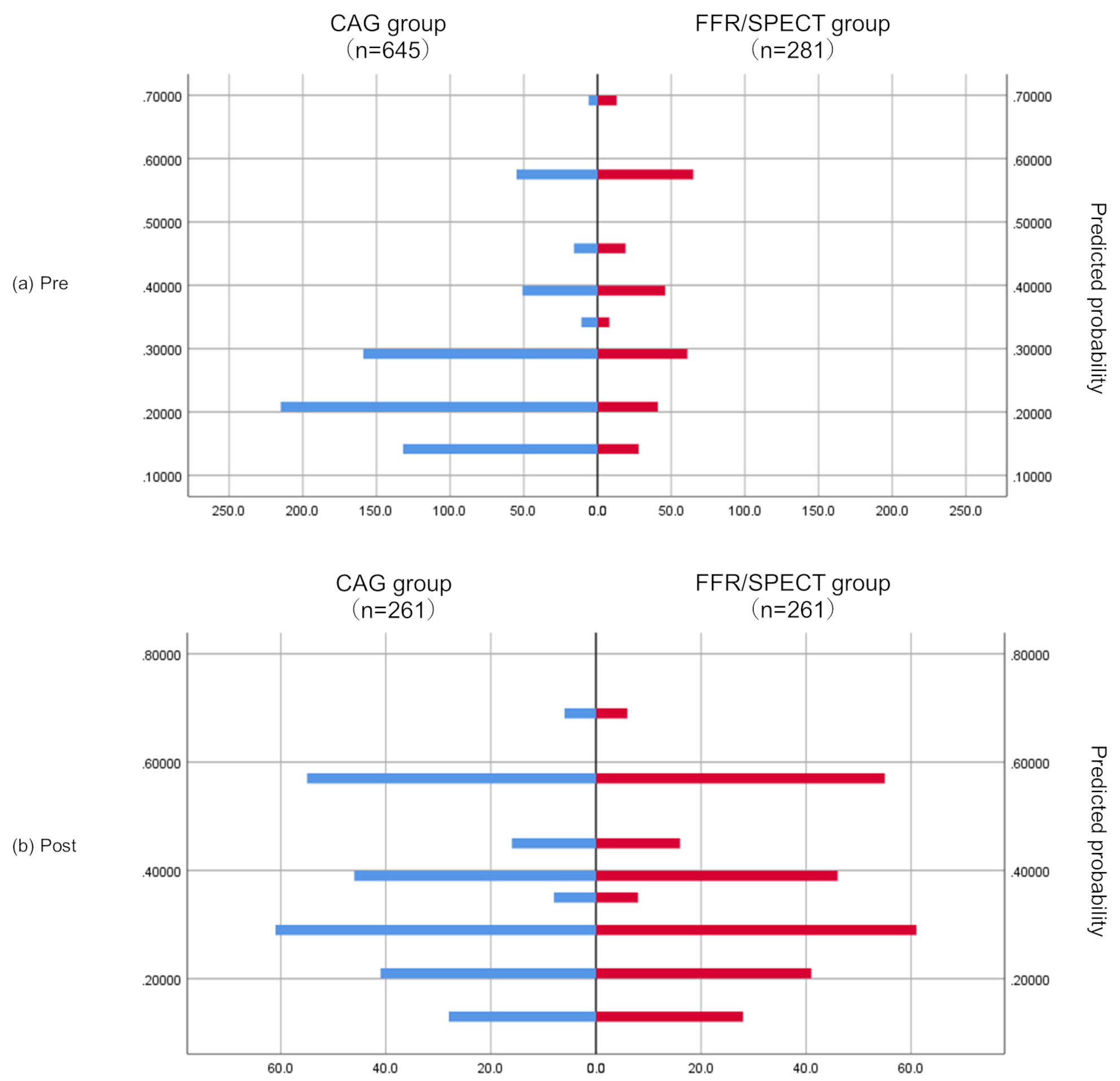

Supplement: Supplementary file 3 — Electronic supplementary material 3 (JPG 203 kb) [file 12350_2020_2502_MOESM3_ESM.jpg]
