# Supplementary material for: Health economics-based verification of functional myocardial ischemia evaluation of stable coronary artery disease in Japan: A long-term longitudinal study using propensity score matching
Source: J Nucl Cardiol. 2021 Jan 18;29(3):1356–69. doi: 10.1007/s12350-020-02502-9 (PMC9162976; doi:10.1007/s12350-020-02502-9)
Supplement: Supplementary file 4 — Electronic supplementary material 4 (PPTX 3885 kb) [file 12350_2020_2502_MOESM4_ESM.pptx]

## Slide 1
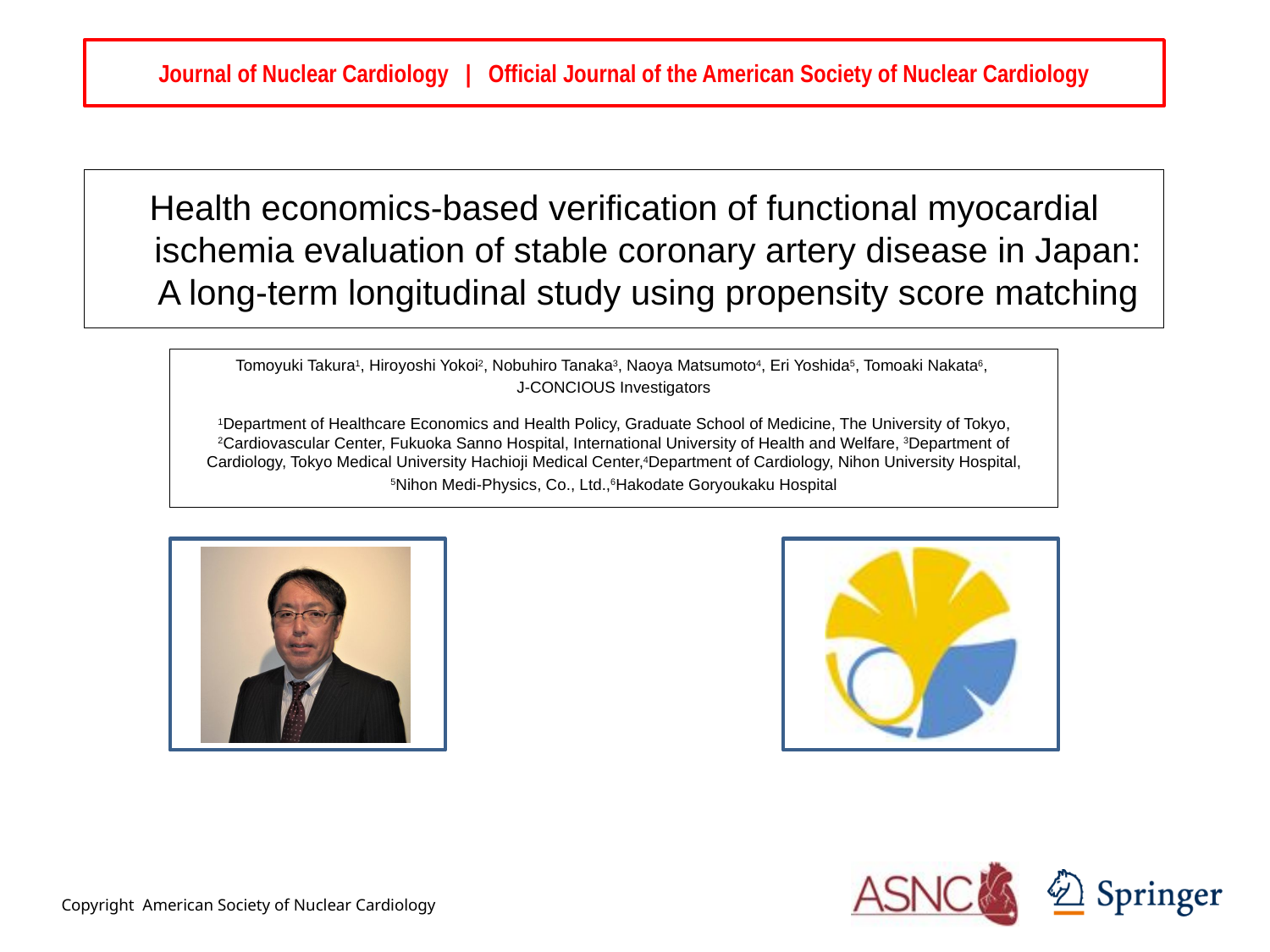

Journal of Nuclear Cardiology | Official Journal of the American Society of Nuclear Cardiology
# Health economics-based verification of functional myocardial ischemia evaluation of stable coronary artery disease in Japan: A long-term longitudinal study using propensity score matching
Tomoyuki Takura1, Hiroyoshi Yokoi2, Nobuhiro Tanaka3, Naoya Matsumoto4, Eri Yoshida5, Tomoaki Nakata6,
J-CONCIOUS Investigators
1Department of Healthcare Economics and Health Policy, Graduate School of Medicine, The University of Tokyo, 2Cardiovascular Center, Fukuoka Sanno Hospital, International University of Health and Welfare, 3Department of Cardiology, Tokyo Medical University Hachioji Medical Center,4Department of Cardiology, Nihon University Hospital,
5Nihon Medi-Physics, Co., Ltd.,6Hakodate Goryoukaku Hospital
Head shot of author
required
Institution
Picture/Logo
Optional
Copyright American Society of Nuclear Cardiology

## Slide 2
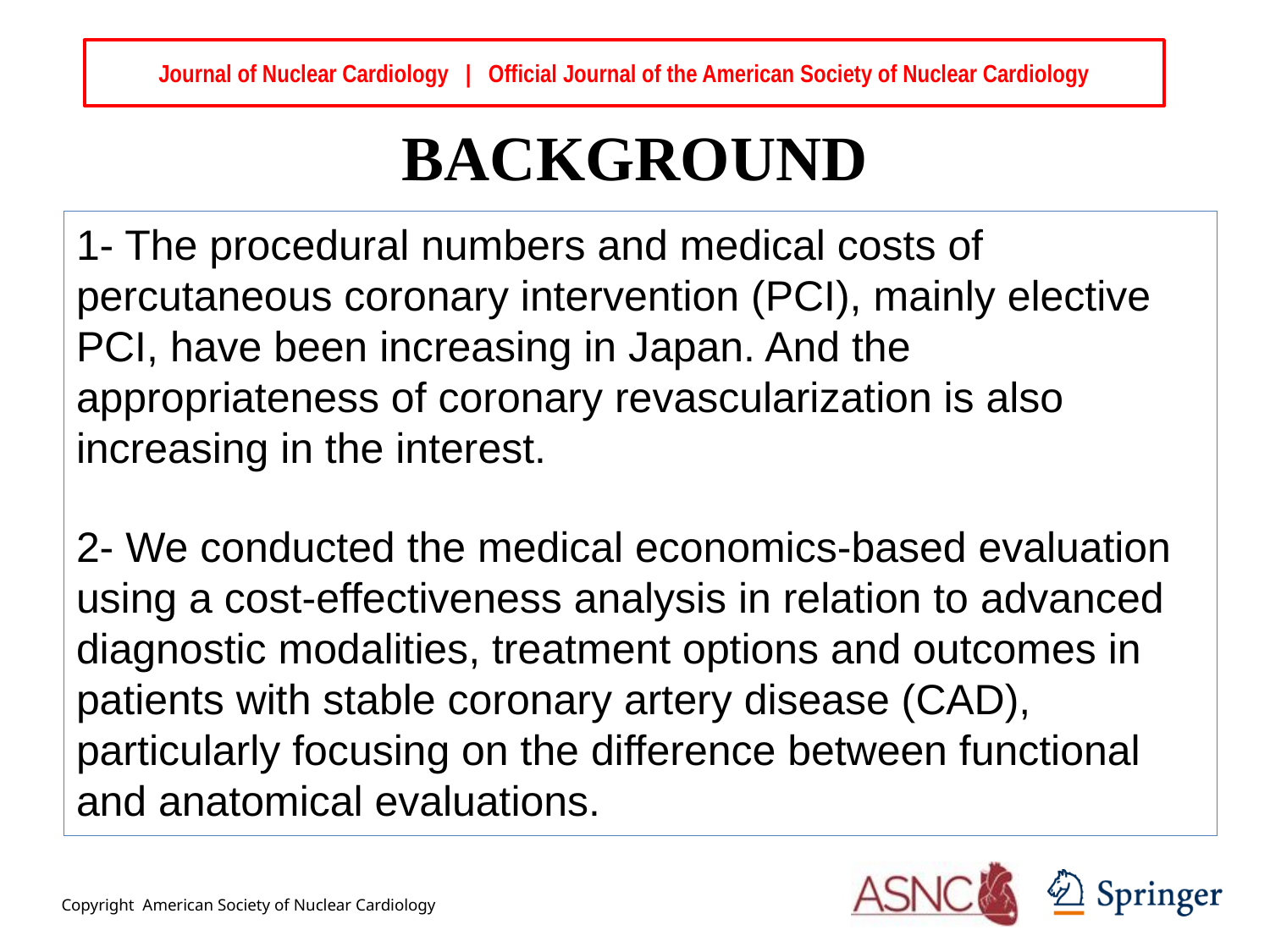

Journal of Nuclear Cardiology | Official Journal of the American Society of Nuclear Cardiology
# BACKGROUND
1- The procedural numbers and medical costs of percutaneous coronary intervention (PCI), mainly elective PCI, have been increasing in Japan. And the appropriateness of coronary revascularization is also increasing in the interest.
2- We conducted the medical economics-based evaluation using a cost-effectiveness analysis in relation to advanced diagnostic modalities, treatment options and outcomes in patients with stable coronary artery disease (CAD), particularly focusing on the difference between functional and anatomical evaluations.
Copyright American Society of Nuclear Cardiology

## Slide 3
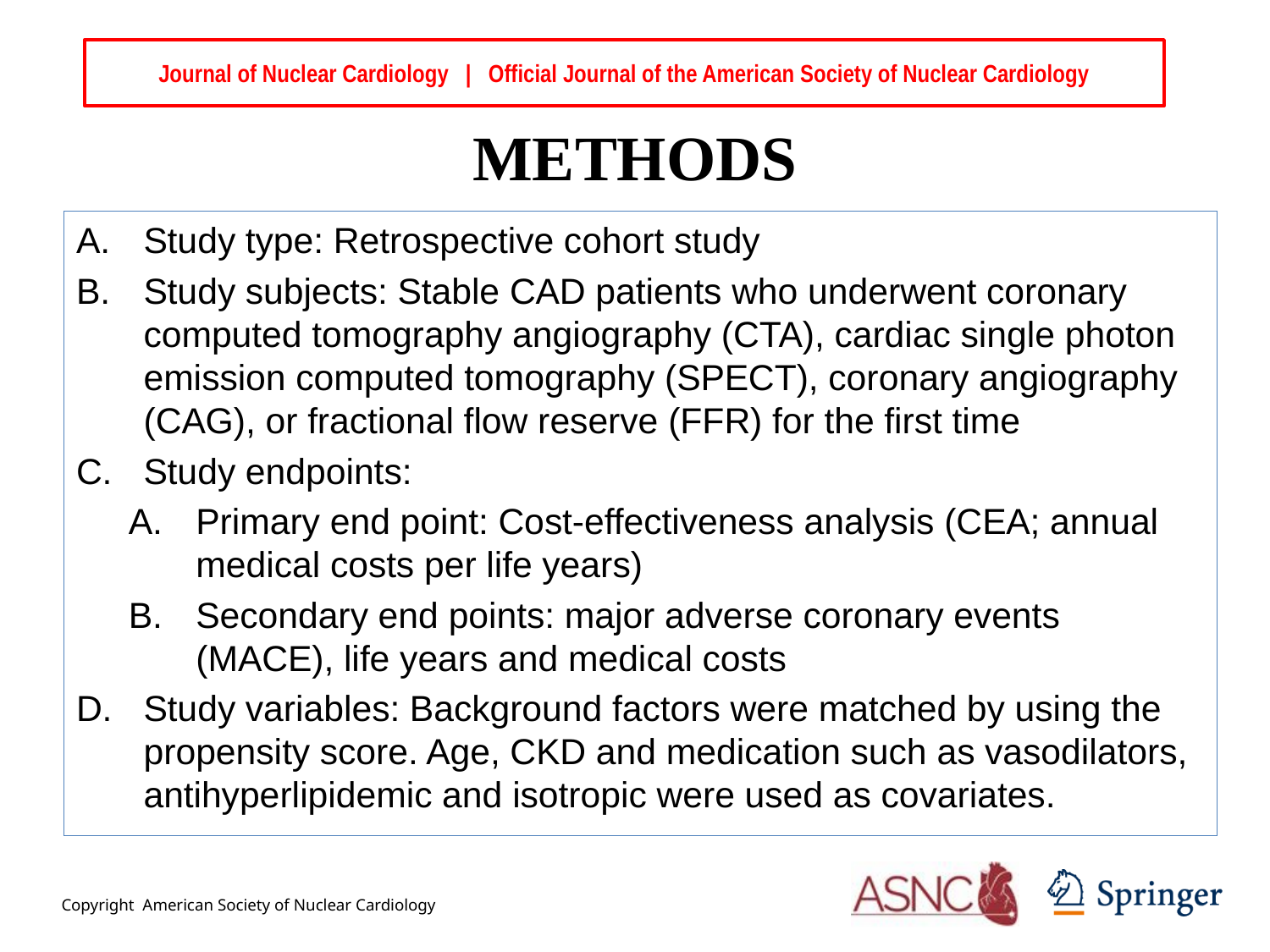

Journal of Nuclear Cardiology | Official Journal of the American Society of Nuclear Cardiology
# METHODS
Study type: Retrospective cohort study
Study subjects: Stable CAD patients who underwent coronary computed tomography angiography (CTA), cardiac single photon emission computed tomography (SPECT), coronary angiography (CAG), or fractional flow reserve (FFR) for the first time
Study endpoints:
Primary end point: Cost-effectiveness analysis (CEA; annual medical costs per life years)
Secondary end points: major adverse coronary events (MACE), life years and medical costs
Study variables: Background factors were matched by using the propensity score. Age, CKD and medication such as vasodilators, antihyperlipidemic and isotropic were used as covariates.
Copyright American Society of Nuclear Cardiology

## Slide 4
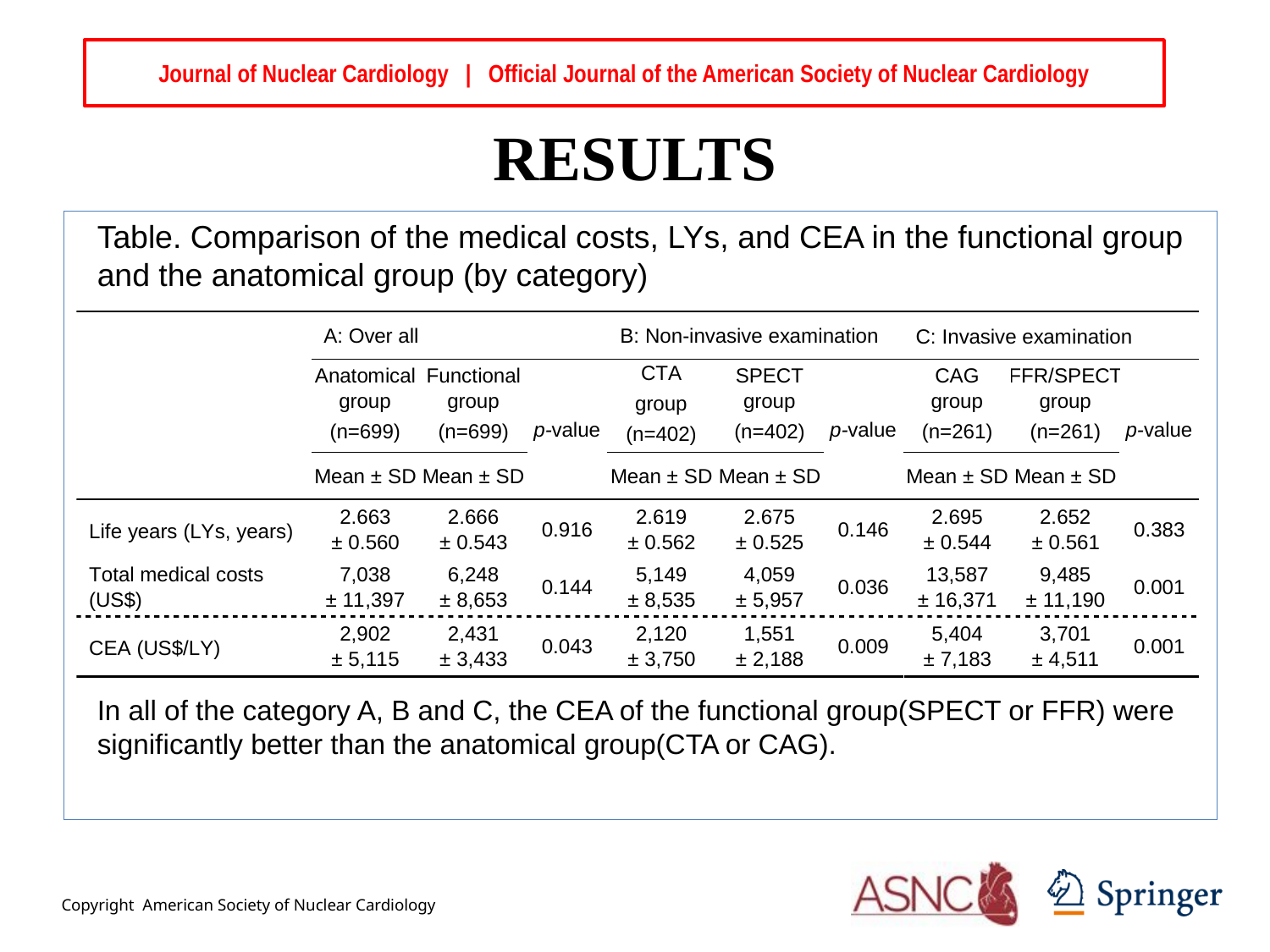

Journal of Nuclear Cardiology | Official Journal of the American Society of Nuclear Cardiology
# RESULTS
Table. Comparison of the medical costs, LYs, and CEA in the functional group and the anatomical group (by category)
In all of the category A, B and C, the CEA of the functional group(SPECT or FFR) were significantly better than the anatomical group(CTA or CAG).
Copyright American Society of Nuclear Cardiology

## Slide 5
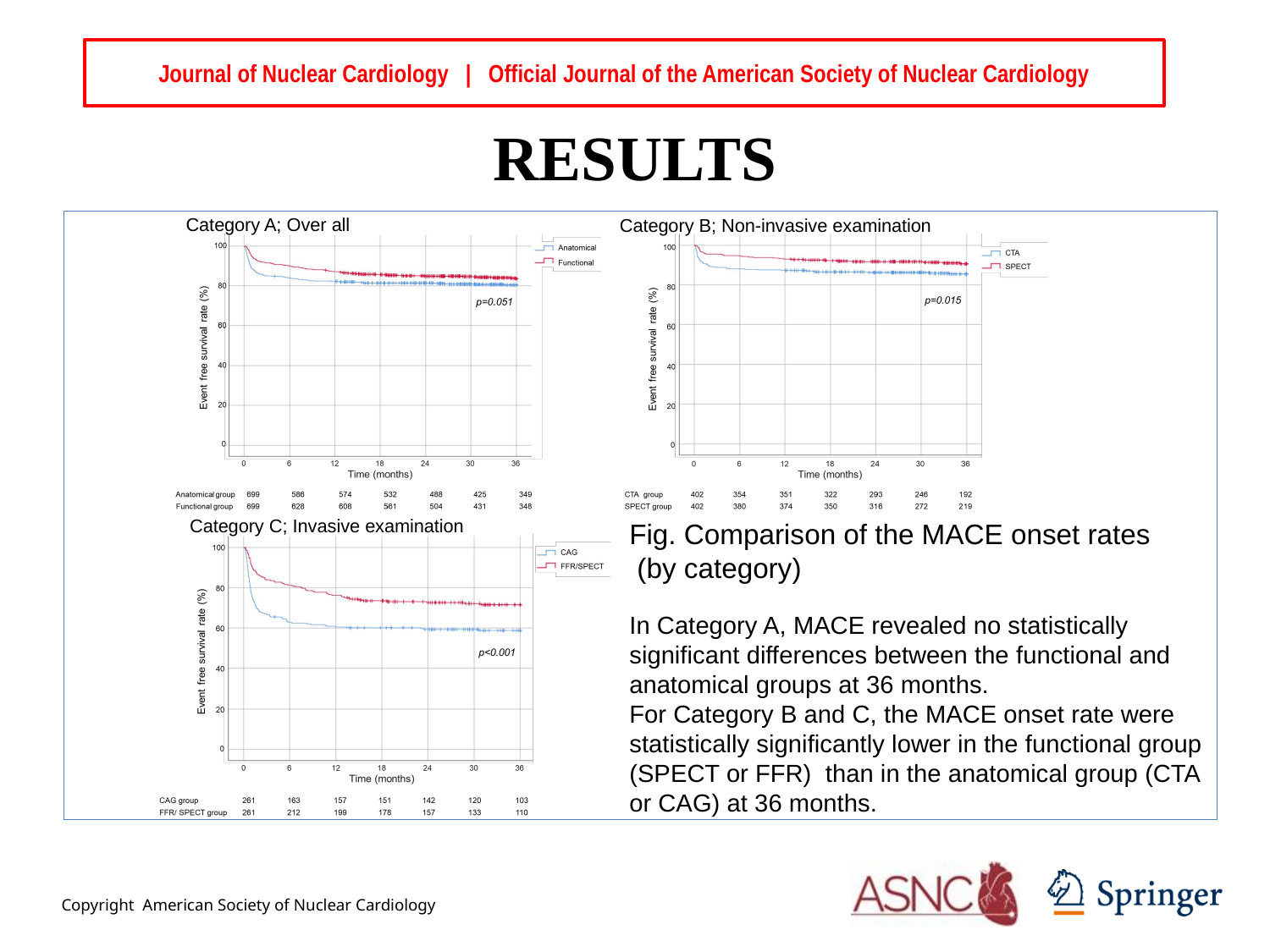

Journal of Nuclear Cardiology | Official Journal of the American Society of Nuclear Cardiology
# RESULTS
Category A; Over all
Category B; Non-invasive examination
Category C; Invasive examination
Fig. Comparison of the MACE onset rates
 (by category)
In Category A, MACE revealed no statistically significant differences between the functional and anatomical groups at 36 months.
For Category B and C, the MACE onset rate were statistically significantly lower in the functional group (SPECT or FFR) than in the anatomical group (CTA or CAG) at 36 months.
<
Copyright American Society of Nuclear Cardiology

## Slide 6
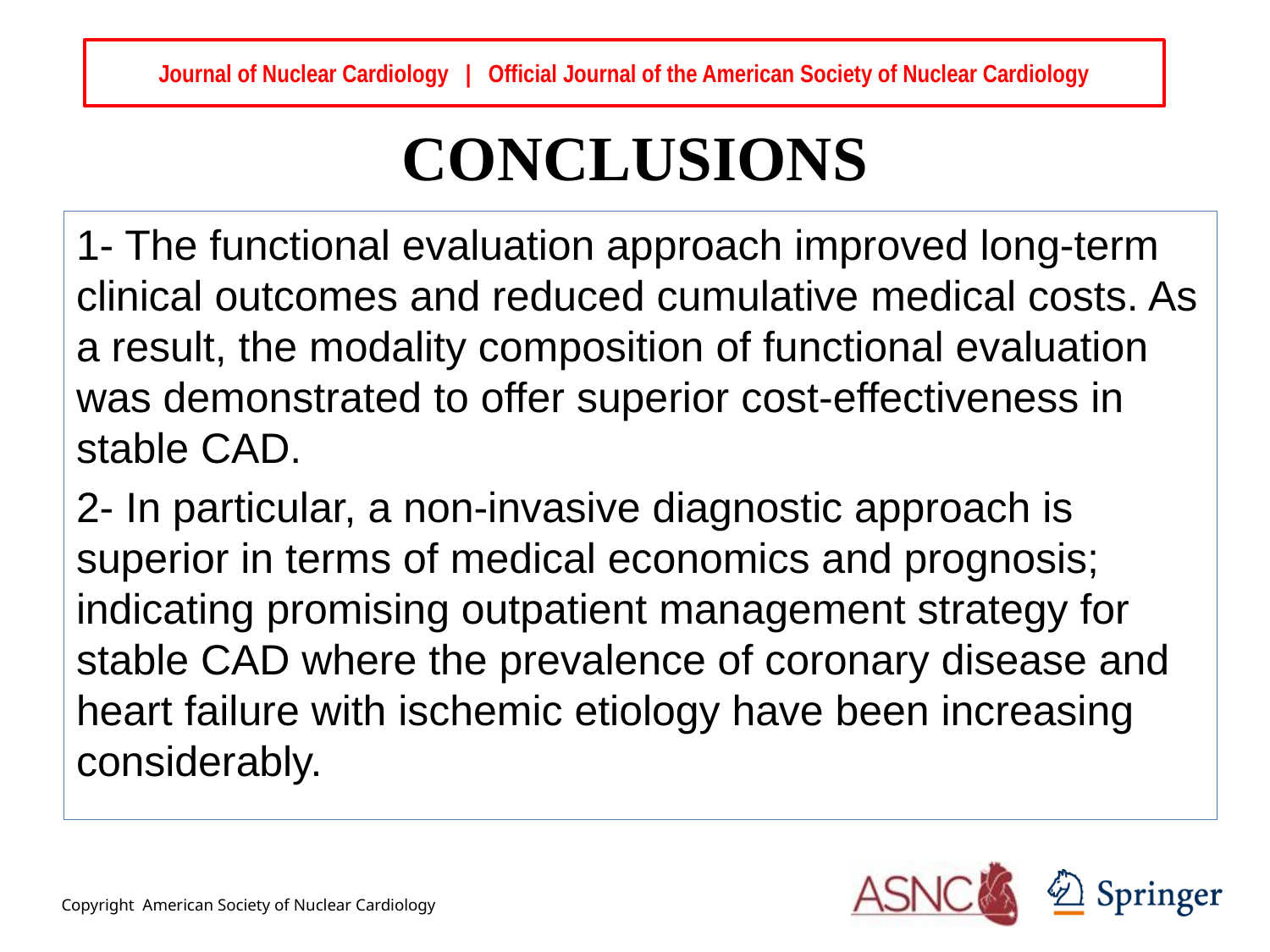

Journal of Nuclear Cardiology | Official Journal of the American Society of Nuclear Cardiology
# CONCLUSIONS
1- The functional evaluation approach improved long-term clinical outcomes and reduced cumulative medical costs. As a result, the modality composition of functional evaluation was demonstrated to offer superior cost-effectiveness in stable CAD.
2- In particular, a non-invasive diagnostic approach is superior in terms of medical economics and prognosis; indicating promising outpatient management strategy for stable CAD where the prevalence of coronary disease and heart failure with ischemic etiology have been increasing considerably.
Copyright American Society of Nuclear Cardiology
